# Antiviral Effectors and Gene Drive Strategies for Mosquito Population Suppression or Replacement to Mitigate Arbovirus Transmission by *Aedes aegypti*

**DOI:** 10.3390/insects11010052

**Published:** 2020-01-12

**Authors:** Adeline E. Williams, Alexander W. E. Franz, William R. Reid, Ken E. Olson

**Affiliations:** 1 Department of Microbiology, Immunology and Pathology, Colorado State University, Fort Collins, CO 80523, USA; adeline.williams@colostate.edu; 2 Department of Veterinary Pathobiology, University of Missouri, Columbia, MO 65211, USA; franza@missouri.edu (A.W.E.F.); reidwi@missouri.edu (W.R.R.)

**Keywords:** *Aedes aegypti*, gene drive, arbovirus, population suppression, population replacement

## Abstract

The mosquito vector *Aedes aegypti* transmits arthropod-borne viruses (arboviruses) of medical importance, including Zika, dengue, and yellow fever viruses. Controlling mosquito populations remains the method of choice to prevent disease transmission. Novel mosquito control strategies based on genetically manipulating mosquitoes are being developed as additional tools to combat arbovirus transmission. Genetic control of mosquitoes includes two basic strategies: population suppression and population replacement. The former aims to eliminate mosquito populations while the latter aims to replace wild populations with engineered, pathogen-resistant mosquitoes. In this review, we outline suppression strategies being applied in the field, as well as current antiviral effector genes that have been characterized and expressed in transgenic *Ae. aegypti* for population replacement. We discuss cutting-edge gene drive technologies that can be used to enhance the inheritance of effector genes, while highlighting the challenges and opportunities associated with gene drives. Finally, we present currently available models that can estimate mosquito release numbers and time to transgene fixation for several gene drive systems. Based on the recent advances in genetic engineering, we anticipate that antiviral transgenic *Ae. aegypti* exhibiting gene drive will soon emerge; however, close monitoring in simulated field conditions will be required to demonstrate the efficacy and utility of such transgenic mosquitoes.

## 1. Introduction

Arthropod-borne viruses (arboviruses), including Zika (ZIKV), dengue (DENV), chikungunya (CHIKV), and yellow fever viruses (YFV), are emerging global health threats that have recently exhibited resurgence in prevalence [1]. The mosquito vector, *Aedes aegypti*, transmits these viruses throughout tropical and sub-tropical regions of the world. Originating from sub-Saharan Africa, *Ae. aegypti* is a highly invasive species that has adapted to urban environments, putting more than 3 billion people at risk of arboviral diseases [1,2]. *Ae. aegypti* females exhibit a high vectorial capacity because they are competent for transmitting medically important arboviruses, have a lifespan exceeding the typical extrinsic incubation periods of most arboviruses, prefer to feed on humans, and are able to ingest multiple bloodmeals during a day [1]. Human vaccines have been developed for only a few mosquito-borne arboviruses (YFV, Japanese encephalitis virus, and DENV), and therapeutic interventions are limited.

Controlling *Ae. aegpyti* populations to limit human exposure to infected vectors is currently the method of choice to prevent arboviral disease transmission. However, vector control relying on insecticide applications is difficult to sustain because of the need for their widespread and long-term deployments, which can select for and increase insecticide resistance in mosquito populations [1,3,4]. Currently, novel mosquito control strategies based on genetic manipulation of mosquitoes are being developed as additional tools to combat disease transmission. Genetic pest management, coined by Fred Gould, encompasses the manipulation of a target organism at the genetic level [5]. In the context of vector control, genetic pest management is aiming at (1) eliminating insect populations by inducing lethality or sterility (population suppression) or (2) replacing wild-type populations with transgenic insects that have been engineered to be resistant to pathogen infection/transmission (population replacement). These techniques may involve the introduction/overexpression (gain-of-function) or disruption (loss-of-function) of gene(s) associated with essential cellular or pathogen pathways, host immunity, sex determination, fecundity, or reproduction. 

## 2. *Aedes aegypti* Population Suppression Strategies

The transgenic *Ae. aegypti* line OX513A (Oxitech, Oxford, UK), has been tested as a RIDL (release of insects carrying a dominant lethal) method in several regions of the world, including the Grand Cayman Islands and Brazil [6,7,8]. These RIDL mosquitoes overexpress a dominant lethal synthetic gene construct, comprised of a tetracycline-repressible transcriptional transactivator (tTAV), which is under the control of a tet operator and a minimal promoter [6]. In the presence of tetracycline, the conformation of tTAV is modified in such a way that it can no longer bind to the tet operator, allowing the OX513A line to be mass produced [7]. In the absence of tetracycline, however, the expression of tTAV causes a feedback overexpression of itself, resulting in lethality at pupation. This technology, however, does not typically lead to 100% adult population suppression (80–95% on average instead [6,7]), allowing migrant *Ae. aegypti* populations to reestablish in the target area. Thus, subsequent releases of transgenic mosquitoes within the target area are required to maintain population suppression over time, which is expensive and laborious. As an alternative, the female-specific flightless line of *Ae. aegypti*, OX3604C, (Oxitech) was tested under large-scale field conditions as another tetracycline-regulated RIDL strain [9]. Homozygous OX3604C transgenic males that mated with wild-type females produced female offspring unable to fly and were thus unable to mate, ingest blood, or reproduce [9]. Male OX3604C mosquitoes displayed significantly fewer fitness deficits than male OX513A mosquitoes, including improved flying speeds and distances [10]. A reason for this phenomenon could be that in the OX3604C line, tTAV production is under the control of a stage- and sex-specific promoter, *AeAct-4*. This promoter may lead to tighter expression of the transgene, which prevents unspecific tTAV overexpression and male lethality; however, the OX3604C line was unable to effectively replace wild-type populations in outdoor cage trials, mostly because of a mating disadvantage associated with the transgenic males [9,10]. 

Previously, it was observed that inoculation of *Ae. aegypti* with the maternally inherited endosymbiotic α-proteobacterium *Wolbachia* profoundly reduced the mosquito’s life span and also restricted arboviral infections in those mosquitoes [11,12,13]. Certain strains of *Wolbachia* are capable of inducing cytoplasmic incompatibility (CI) in both naturally infected and trans-infected mosquitoes. In that case only females carrying the endosymbiont can produce viable offspring after mating with infected or naïve males, but *Wolbachia*-infected males are unable to reproduce after mating with uninfected females [14]. Generally, *Ae. aegypti* is not considered a natural host for *Wolbachia*. Thus, the endosymbiont needs to be adapted to new hosts such as *Ae. aegypti*. *wMelPop*, a life-shortening strain of *Wolbachia* naturally found in *Drosophila melanogaster*, was transfected into mosquito cell lines including an *Ae. aegypti* cell line. *Wolbachia wMelPop* was then passaged in those mosquito cell lines for several years prior to injection into *Ae. aegypti* mosquitoes, thereby creating a less-pathogenic variant, *wMelPop*-CLA [15]. However, *wMelPop*-CLA was still found to be life-shortening for trans-infected *Ae. aegypti*, while other *Wolbachia* strains such as *wMel* imposed minimal-to-no fitness costs to the same mosquito species [16,17]. Importantly, both *Wolbachia* strains antagonized DENV in the mosquito. From 2014 onwards, more than 4 million *Wolbachia wMel* trans-infected *Ae. aegypti* were released in Townsville, Australia (population: ~187,000) over a two-year period [18]. Mosquito releases continued until, for two consecutive weeks, local *Ae. aegypti* population samples persistently displayed a *Wolbachia* frequency exceeding 50%. Eventually, these releases led to the effective spread of *Wolbachia* into local *Ae. aegypti* populations as well as a significant reduction in locally acquired dengue cases—dropping from a median of >130 cases before the trial to 4 cases four years after the trial was concluded [18]. Similar trials are currently deployed in Yogyakarta, Indonesia, Rio de Janeiro, Brazil, and Medellin, Colombia, where mosquito population structure and overall dengue incidence all vary [18]. 

While *Wolbachia* holds promise for *Ae. aegypti* control, there are mosquito-attributed genetic component(s), including genes involved in cell adhesion or cytoskeleton integrity, that influence the efficacy of *Wolbachia*-mediated viral blocking [19]. Therefore, the efficacy of this strategy may be limited in dynamic mosquito populations [19]. However, *Wolbachia*-infected mosquitoes that exhibit “weak” viral blocking (i.e., they are still susceptible to arboviruses) exhibit heightened fitness costs as compared to *Wolbachia*-infected mosquitoes that exhibit strong viral blocking [19]; therefore trans-infected mosquitoes exhibiting high levels of *Wolbachia*-mediated virus blocking could have a fitness advantage over trans-infected mosquitoes that are still susceptible to viruses.

## 3. Antiviral Effector Strategies for *Aedes aegypti* Population Replacement

Compared to population suppression strategies, implementation of population replacement strategies in the field have been delayed because pathogen resistance is difficult to identify, engineer, or select for. As early as 1991, it was observed that certain *Ae. aegypti* strains naturally harbored arbovirus resistance genes that could be transferred to susceptible mosquito populations through selective breeding [20,21]. Quantitative trait loci (QTL) experiments later identified regions of the *Ae. aegypti* genome associated with arbovirus resistance [22]. Specifically, the *dcr2* gene was found to be associated with *Ae. aegypti* resistance to dengue 2 virus (DENV2), suggesting RNAi is playing a role in mosquito antiviral immunity [23]. The small-interfering RNA (siRNA) pathway is the best characterized antiviral immune mechanism in *Ae. aegypti* [24]. Briefly, viral dsRNA intermediates are recognized by mosquito Dicer-2 and processed into 21 base-pair (bp) siRNA duplexes. These siRNA duplexes interact with Dicer-2 and R2D2 to be unwound. One of the strands (“guide strand”) is incorporated into the RNA-induced silencing complex (RISC) containing the endonuclease Argonaute-2. The 21 nucleotide (nt) guide strand directs RISC to complementary (viral) RNA sequences in the cell cytoplasm. Argonaute-2 of RISC then cleaves the targeted RNA. 

As early as 2006, the antiviral siRNA pathway of *Ae. aegypti* was exploited to engineer transgenic pathogen-resistant mosquitoes [25]. These mosquitoes expressed from the *carboxypeptidase A (AeCPA)* bloodmeal-inducible promoter an inverted-repeat (IR) RNA derived from the DENV2 prM encoding RNA sequence. The transcribed IR, mimicking a fragment of the viral dsRNA intermediate, was processed by the mosquito’s RNAi machinery into DENV2-specific siRNAs, causing destruction of the viral RNA in infected midgut cells. Fourteen days post-infection (dpi), 96% of the transgenic mosquitoes did not exhibit any level of DENV2 infection and consequently did not transmit the virus in in vitro transmission assays [25]. This same strategy has been used in transgenic *Ae. aegypti* expressing the same IR from the salivary gland-specific *Ae30K b* promoter [26], and in an improved homozygous line (“Carb109M”), where the IR, again under control of the *AeCPA* promoter, was expressed from a more stable genetic locus [27]. These mosquitoes continued to be nearly 100% resistant to DENV2 through >50 generations as compared to their wild-type counterparts and displayed minimal fitness costs [27]. Conserved, efficacious RNA sequences spanning the NS2b-NS4a regions of the ZIKV genome have also been identified, which could be used to construct ZIKV targeting IRs [28].

Another antiviral effector strategy that has been explored makes use of ribozymes, which are short RNA molecules that have enzyme-like capabilities to cleave RNA molecules [29]. Ribozymes are found in all domains of life and have the capacity to self-cleave (e.g., group I and II introns) or to cleave RNA at specific sites (e.g., hammerhead and hairpin ribozymes) [29]. Ribozymes can be engineered to function *in trans* to cleave a target RNA, and several types have been investigated as antiviral mediators in *Aedes* spp. cell lines and transgenic *Ae. aegypti* mosquitoes [30,31,32]. For example, an *Ae. albopictus* cell line (C6/36) was transformed to express “dual” trans-splicing group 1 introns that targeted both DENV and CHIKV [31]. These group 1 ribozymes targeted the highly conserved 5′ and 3′ cyclization sequences of DENVs and a highly conserved region in the *CHIKV NS1* gene. Additionally, these ribozymes were designed to express a ΔN-Bax 3′ exon upon splicing of the viral genome, which caused the induction of cell apoptosis. Thus, the ribozyme led to the destruction of the arbovirus *per se* as well as the infected cell. All cell lines transfected with these ribozymes completely suppressed both DENV1-4 and CHIKV replication in vitro [31]. Because the ribozymes target highly conserved sequences, this strategy could be a promising method to develop transgenic mosquitoes resistant to multiple arboviruses; furthermore, targeting highly conserved viral sequences could limit the viruses’ abilities to develop resistance against the effector transgene. Transgenic *Ae. aegypti* expressing a hammerhead ribozyme (hRZ) were recently developed targeting the attenuated CHIKV strain 181/25 [32]. In principal, hammerhead ribozymes target small (15–16 nt) sequences, are active without relying on the host-cell machinery, and are stable at a wide variety of temperatures [32]. Seven hRZs were designed to target the CHIKV structural polyprotein-encoding region of the viral RNA genome, and each individual hRZ completely inhibited CHIKV 181/25 in cell culture. Importantly, six out of seven transgenic *Ae. aegypti* lines engineered to express a CHIKV 181/25 hRZ completely inhibited CHIKV replication in the salivary glands at 7 dpi [32].

Transgenic *Ae. aegypti* have also been engineered to overexpress genes involved in the conserved, antiviral-immune Janus kinase (JAK)-signal transducer and activator of transcription (STAT) pathway [33]. A cytokine signaling pathway in mammals, the Dipteran JAK/STAT pathway is activated by ligand binding to and dimerization of the transmembrane receptor Domeless (Dome) (or its species-specific orthologs) [34]. Associated JAKs, such as the Hop kinase, self-phosphorylate, then phosphorylate the Dome receptor, and form docking sites for other STAT proteins; following their phosphorylation, the STATs are translocated into the nucleus where they serve as transcription factors for several antiviral restriction factors [34]. Transgenic mosquitoes were engineered to overexpress either the Dome receptor or the Hop kinase, as well as to overexpress both elements simultaneously [33]. Reduction of DENV2 and DENV4 infections clearly varied among individual transgenic mosquitoes; therefore, this transgenic approach may not be sufficient to completely block virus transmission. Curiously, the transgenic mosquitoes exhibited a significant reduction in fecundity as compared to controls. Furthermore, overexpression of the JAK/STAT pathway in these mosquitoes did not effectively antagonize ZIKV or CHIKV [33]. 

*Ae. aegypti* expressing a cluster of small synthetic DENV3 and CHIKV-targeting miRNAs were engineered to suppress both viruses by way of the miRNA pathway [35]. Similar to the siRNA pathway, the miRNA pathway is highly conserved and uses miRNAs as guides for Argonaute-1 mediated sequence-specific degradation. However, pri-miRNAs (instead of long dsRNA, as in the siRNA pathway) are processed by Drosha in the nucleus, exported by Exportin5 into the cytoplasm, and processed by Dicer-1 into 21–25 nt miRNAs, which are then loaded into miRNA induced silencing complexes (miRISCs). Eventually, this then results in a sequence-specific degradation of mRNAs or translational gene silencing [35]. Exploiting this pathway, four effector constructs were tested in transgenic mosquitoes: two lines expressing four or six miRNAs complementary to DENV3 or CHIKV RNAs, respectively, both under control of the constitutively expressing *polyubiquitin* (*AePUb*) promoter and two lines expressing ten and three miRNAs targeting both DENV3 and CHIKV RNAs, respectively, either from the *AePUb* or the *AeCPA* promoter [35]. Transgenic mosquitoes engineered to target either DENV3 or CHIKV (but not both) showed elevated levels of resistance to their viral target, while those mosquitoes engineered to target both viruses simultaneously were significantly less susceptible to DENV3 (~10% infection prevalence), but were unable to affect CHIKV replication [35]. These results suggest that DENV3 may be more vulnerable to miRNA-mediated silencing than CHIKV. Also, several of the transgenic lines exhibited heightened fitness costs. This same miRNA-based strategy was used to engineer *Ae. aegypti* targeting multiple ZIKV strains (Cambodian FSS13025 and Puerto Rican PRVABC59) [36]. These mosquitoes expressed a polycistronic cluster of eight synthetic ZIKV-targeting miRNAs, although only five of them were properly processed. Nonetheless, heterozygote mosquitoes of the transgenic line significantly reduced ZIKV titers by >2 logs plaque-forming units per milliliter (PFU/mL) whereas homozygote mosquitoes of the line completely inhibited ZIKV midgut infection and dissemination at 4 and 14 dpi. Further, these transgenic mosquitoes completely lacked virus in saliva as shown by transmission assays [36].

Recently, transgenic *Ae. aegypti* were engineered to express single-chain antibodies to resist DENV infection [37]. Based on a monoclonal antibody (1C19) derived from human patient IgGs, Buchman and colleagues (2019) developed a homozygous transgenic line of *Ae. aegypti* that expresses a broadly neutralizing, single-chain variable fragment targeting all four DENV serotypes [37]. This single-chain antibody recognizes the BC loop of domain II in the envelope protein and was optimized for expression in mosquitoes [38]. Remarkably, these mosquitoes have shown to be resistant to all four DENV serotypes, displaying a complete lack of virus infection in midguts, carcasses, and saliva at 14 dpi as confirmed by plaque assays or quantitative RT-PCR [37]. The transgenic mosquitoes displayed low fitness costs as compared to wild-type mosquitoes, although the female median survival rate was significantly lower among the transgenic mosquitoes [37]. Nevertheless, this is a pivotal study as it demonstrates the development of transgenic mosquitoes engineered to simultaneously resist all four DENV serotypes. Table 1 lists all transgenic *Ae. aegypti* lines that have been engineered to date to be arbovirus resistant.

In support of a population replacement strategy, it is becoming apparent that *Ae. aegypti* genetically modifies itself. Modern genomic sequencing and bioinformatics approaches have detected numerous examples of DNA sequences derived from non-retroviral RNA virus genomes integrated into insect genomes. These pathogen-derived DNAs are apparently a product of retrotransposons in the vector that encode RNA-dependent DNA polymerases (reverse transcriptases) and convert RNA viral genomes into short DNA sequences creating retrotransposon-viral hybrid sequences integrated into the vector’s genome [39]. Non-retroviral integrated RNA virus sequences (NIRVS) occur at relatively high frequency in the genomes of the arboviral vectors *Ae. aegypti* and *Ae. albopictus*, are not distributed randomly and contribute to mosquito antiviral immunity [39,40,41]. 

Successful population replacement strategies involving anti-pathogen effectors requires either that the engineered, resistant population would supplant the wild-type population of *Ae. aegypti*, or that the transgene would be driven through the targeted wild-type population. The latter scenario would require the application of a gene drive system, which needs to be linked to the antiviral effector gene. However, until now, such a system has not been made available. Until such lines are ready for testing, the efficacy of *Ae. aegypti* population replacement strategies under more relevant field conditions remains unknown.

## 4. Gene Drive Principles

**Figure 1:**
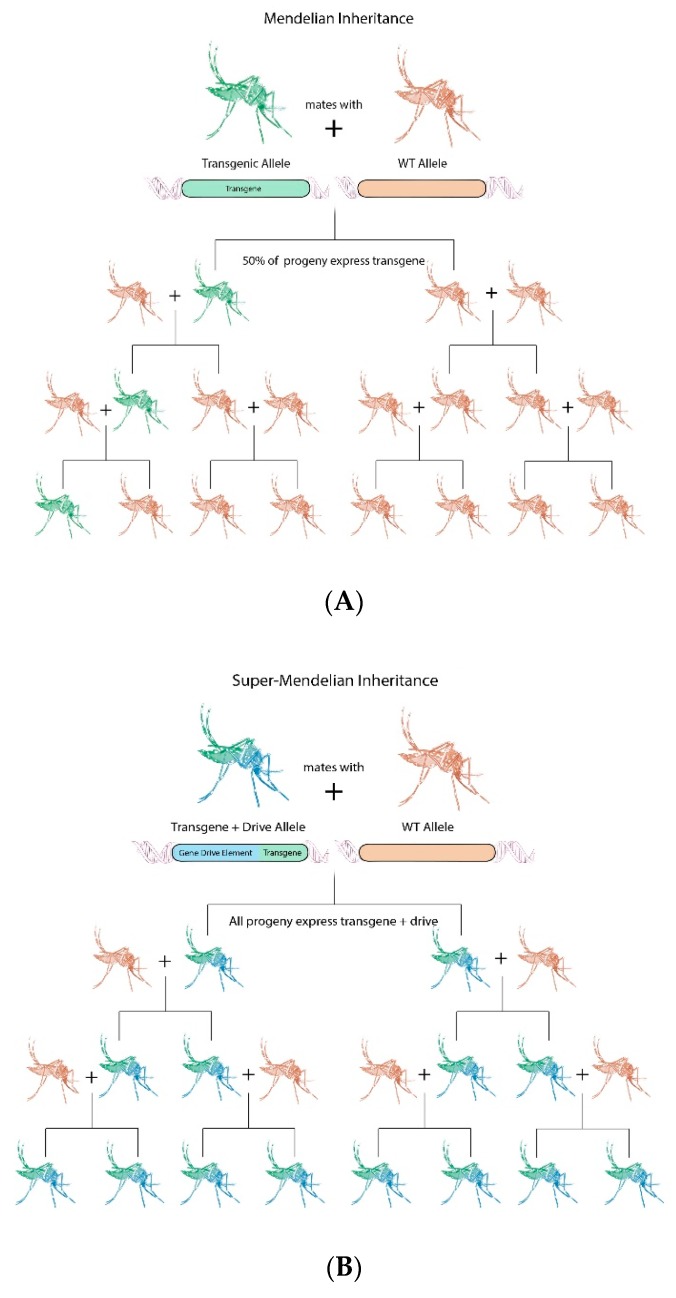
Mendelian versus “Super” Mendelian inheritance. (**A**) Mendel’s law of independent assortment predicts an inheritance rate of 50% for a transgene when it is not sex-linked. Without repeated introduction, loss of the transgene is expected because of multiple factors including genetic drift and fitness cost of the transgene. (**B**) Homing endonuclease based gene drives supersede Mendel’s law of independent assortment by converting wild-type alleles into gene drive bearing alleles in the germline. This then leads to fixation of the gene drive in the target population.

The use of transgenes to confer arboviral resistance may carry unintended fitness costs, ultimately leading to their loss in wild-type populations. Coupling the effector to a gene drive system can overcome fitness costs by pushing the transgene through the population at levels higher than expected from typical Mendelian inheritance. Gene drive, or meiotic drive, is the super-Mendelian inheritance pattern of a selfish genetic element (SGE) that allows it to rapidly spread through populations, even if it does not improve the survival or reproduction of its host [42] (Figure 1). In other words, gene elements that are inherited from a parent with an allele frequency of >50% are considered to exhibit gene drive [42].

Gene drives occur naturally in many organisms, including several insect species such as *D. melanogaster* and *Ae. aegypti*. In the former, for example, heterozygous males that carry one copy of the mutant *Segregation distorter* (*Sd*) gene and one copy of the corresponding *Responder* (*Rsp*) gene will transmit *Sd* to 100% of the progeny (instead of the expected 50%) [42]. This occurs because *Sd* prevents the homologous *Rsp*-bearing chromosome from properly condensing, which causes the chromosome to self-destruct. *Rsp* sperm therefore do not fully develop, and only *Sd*-mutant sperm can reach maturity and are passed to progeny [42].

Sex ratio distortion occurring in certain *Ae. aegypti* populations has also been linked to a natural gene drive system, although the underlying mechanisms are not well understood [43,44,45]. A recessive female-determining allele (mm) or the dominant male-determining M allele counterpart is linked to a gene drive-like element (a meiotic drive allele, M^D^) on chromosome 1. M^D^ causes breakage of the homologous m-bearing chromosome, which leads to the development of male-biased populations [46]. This phenomenon is sometimes referred to as male meiotic drive [46,47]. The system is germline-linked, allowing vertical transmission of the gene drive element to the offspring. A recent study identified 209 genes associated with this endogenous drive system, many of which were involved in the glycosylphosphatidylinositol (GPI)-anchor biosynthesis pathway or exhibited general protein translation and/or regulatory functions. These observations suggest that the endogenous drive system is highly complex since it is associated with essential proteins that have broad regulatory functions [45]. Many GPI-anchored proteins interact with the Ras signaling pathway, which could serve as a mediator for meiotic drive since this pathway is involved in cell proliferation [45].

SGEs encompass meiotic drive chromosomes, transposable elements, sex-ratio distorting elements, gametic killers, and homing endonuclease genes (HEGs) [48,49]. All of these elements distort Mendelian transmission patterns through different mechanisms. SGEs can alter the proportion of homologous allele transmission during meiosis (meiotic drivers) or increase their accumulation over generations via replication in the germline (transposable elements). SGEs can also selectively disrupt sex chromosome maturation through “killer” alleles that are only expressed on specific sex chromosomes (sex ratio distorters); alternatively, they can induce a double-stranded break (DSB) at a specific target site of the genomic DNA to insert a copy of the genetic element into the target site (homing). All of the aforementioned mechanisms (except transposable elements) convert wild-type alleles into transgene bearing alleles carrying the gene drive element at its specific target site [49,50,51,52].

SGEs can be genetically linked to exogenous genes-of-interest (g.o.i.) such as antiviral effectors to allow for super-Mendelian inheritance of the effector gene even if it carries a fitness load for the organism. However, the design and application of antiviral effectors in combination with SGEs to convert pathogen-susceptible wild-type *Ae. aegypti* populations into pathogen-resistant populations requires several important aspects for consideration. These systems need to be heritable and robustly transmitted to the subsequent generations of the target population. Effective gene drive systems must have the following attributes [53]:

(1) Compensate for any loss of fitness associated with the effector gene;
(2) Link tightly to complex effector genes that are associated with a fluorescent marker;
(3) Drive the effector gene relatively quickly to fixation within the target population;
(4) Adapt to genetically diverse strains of mosquitoes;
(5) Remain confined to the targeted species irrespective of population structure and mating dynamics between species;
(6) Resist mutations that diminish or block drive to be sustained in nature;
(7) Be socially accepted by those communities who might benefit.

## 5. Gene Drive Technologies for *Aedes aegypti*

Several SGEs have been considered and adapted for the use in *Ae. aegypti*. For example, maternal-effect dominant embryonic arrest, or Medea, are elements that cause lethality in all progeny that did not inherit the Medea element [54]. Medea acts as a threshold-dependent gene drive system when transgenic insects expressing the element are released in high numbers [54,55]. For example, transgenic *D. melanogaster* were engineered to express two polycistronic microRNAs that, under the control of the maternal germline-specific *bicoid* promoter, silence the *myd88* gene [54]. Silencing *myd88*, a gene involved in dorsal-ventral patterning, results in lethality. The transgenic flies also expressed from the zygotic *bottleneck (bnk)* promoter a maternal rescue (‘antidote’) copy of the *myd88* gene, which has been modified in such a way that it could not be targeted by the two microRNAs of the killer component. As a consequence, only progeny containing the synthetic antidote (along with the killer component) survived [54]. Although genes that could serve as targets for a Medea-like system have been identified in *Ae. aegypti*, further refinement to optimize spatial and temporal expression of the antidote and killer components is needed [50].

**Figure 2:**
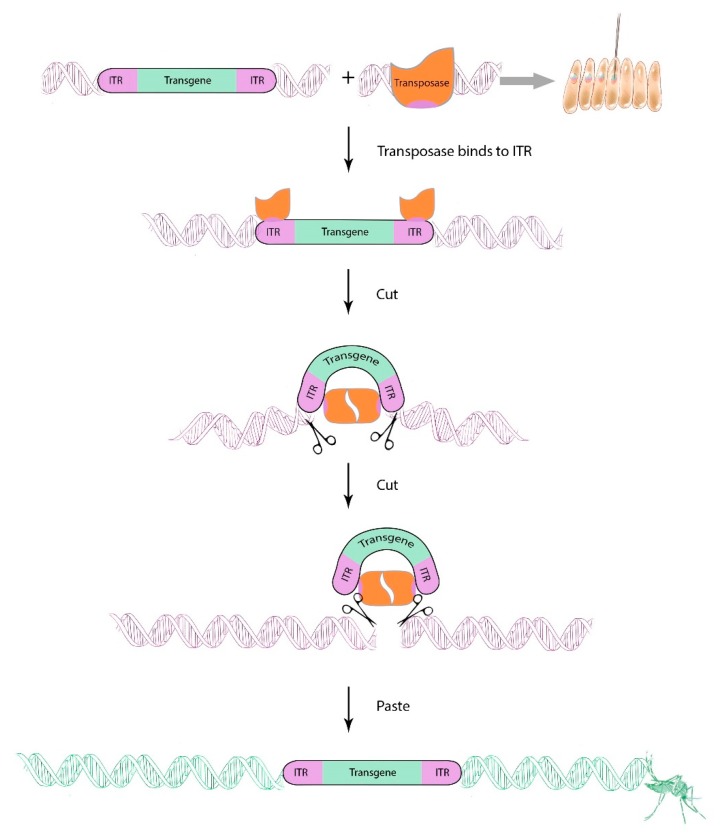
Non-autonomous class II transposable elements can be used to generate transgenic *Aedes aegypti*. Two DNA plasmids are co-injected into pre-blastoderm embryos: (1) A plasmid encoding the gene-of-interest (g.o.i.), which is flanked by inverted terminal repeat (ITR) sequences that serve as binding sites for the transposase; (2) another “helper” plasmid encoding the transposase. Once the transposase is transcribed and expressed, it binds to the ITR region of the g.o.i.-bearing construct. Two transposase units dimerize, induce a DSB, and cleave the transgene (including the ITRs) out of the plasmid. The transgene-transposase complex then binds and inserts the transgenic cargo at defined short recognition sequence motifs into the mosquito genome.

Transposable elements have been used to introduce exogenous genetic material into *Ae. aegypti* as early as 1989 [56], although their utility as gene drive elements is limited. Autonomous transposable elements are SGEs that can remobilize following their integration into the host genome and increase their copy number during remobilization; non-autonomous transposable elements, on the other hand, require an externally provided transposase and, consequently, cannot re-mobilize on their own. Several Class II DNA transposable elements have since been characterized and used in *Ae. aegypti* as non-autonomous gene insertion vectors, including *piggyBac* [57] (derived from a *Trichoplusia ni* cell line [58]), *Hermes* [59] (derived from *Musca domestica* [60]), and *mariner Mos1* [61] (derived from *D. mauritiana* [62]). These elements have been extensively described [63]. *piggyBac* and *mariner Mos1* are the most commonly used non-autonomous transposons to generate transgenic *Ae. aegypti* [64]. A DNA plasmid-based transgene containing a g.o.i. can be flanked by the short inverted terminal repeats (ITR) of the transposon serving as binding sites for the homologous transposase, which is supplied by a separate helper plasmid (Figure 2). Once co-expressed in the preblastoderm mosquito embryo, the transposase binds to both ITRs of the g.o.i. construct and cleaves the construct before integrating it into the *Ae. aegypti* genome in a cut-and-paste manner. 

Initial enthusiasm for transposable elements as gene drive mediators stemmed from their presumed ability, when autonomous, to spread within their host genome, which could consequently drive any linked effector gene(s) at a high rate. Class II DNA transposable elements, however, have since lost their appeal in this regard because they cannot efficiently remobilize in *Ae. aegypti* [65]. Furthermore, transposable elements insert themselves into the genome quasi-randomly, which may include unstable genome loci. This then can lead to positional variegation of the transgene or undesirable fitness costs to the target organism.

HEGs, on the other hand, have the significant advantage of site specificity [66,67]. For example, the I-*Sce*I mega-endonuclease was originally isolated from yeast mitochondria in an intronic region of the 21S rRNA gene and was shown to integrate itself into specific regions of intron-minus forms of the gene, converting them into intron-plus alleles [68]. I-*Sce*I recognizes and cleaves a specific 18 bp sequence motif, which leaves a short 5′ overhang and creates a DSB, serving as a target site for the first proof-of-concept synthetic HEG-based gene drive system in insects [69,70]. The DSB is repaired by homology-directed repair (HDR) using the HEG containing homologous chromosome as a template. Notably, mosquito genomes encode many HEG-specific sequence motifs, some of which may reside in unfavorable loci; this could cause the HEG to become an unpredictable gene drive mediator. Zinc-finger nucleases (ZFNs) [71] and transcription activator-like effector nucleases (TALENs) [72] can be engineered to act as homing endonucleases by designing their target recognition sequences so that they recognize a specific endogenous gene sequence in the organism-of-choice. However, both ZFNs and TALENs are difficult to engineer, and while TALENs are cheaper to custom-engineer than ZFNs, they became overshadowed by the CRISPR/Cas9 system, introduced in 2012 [73].

The CRISPR/Cas9 system was first used in *Ae. aegypti* in 2015 [74], employing the Cas9 protein from *Streptococcus pyogenes*. Cas9 is part of the bacterial immune response to phages and is capable of introducing site-specific DSBs in the genomes of diverse species when programmed with a cognate guide (g)RNA containing crRNA/tracrRNA molecule(s). The Cas9 effector complex identifies a specific sequence immediately downstream of the target, termed the protospacer adjacent motif (PAM), in the host genome. A 17–20 bp stretch of the gRNA binds to the PAM site, thereby stabilizing the Cas9/gDNA complex, which results in a DSB cleavage three base pairs upstream of the PAM. Owing to the simplicity of the required PAM sequence, it is predicted that, on average, a *S. pyogenes*-Cas9 PAM is present in the *Ae. aegypti* genome once every 17 base pairs [75]. Following a DSB, the cell must repair the genomic DNA molecule, which largely occurs through non-homologous end joining (NHEJ) or through HDR. In somatic tissues and in the absence of a homologous DNA template, NHEJ is the likely means of DNA repair, while in the germline, HDR is the more likely DNA repair mechanism helping to conserve genome integrity. Methods and applications for CRISPR/Cas9 editing in *Ae. aegypti* are outlined in [75,76].

**Figure 3:**
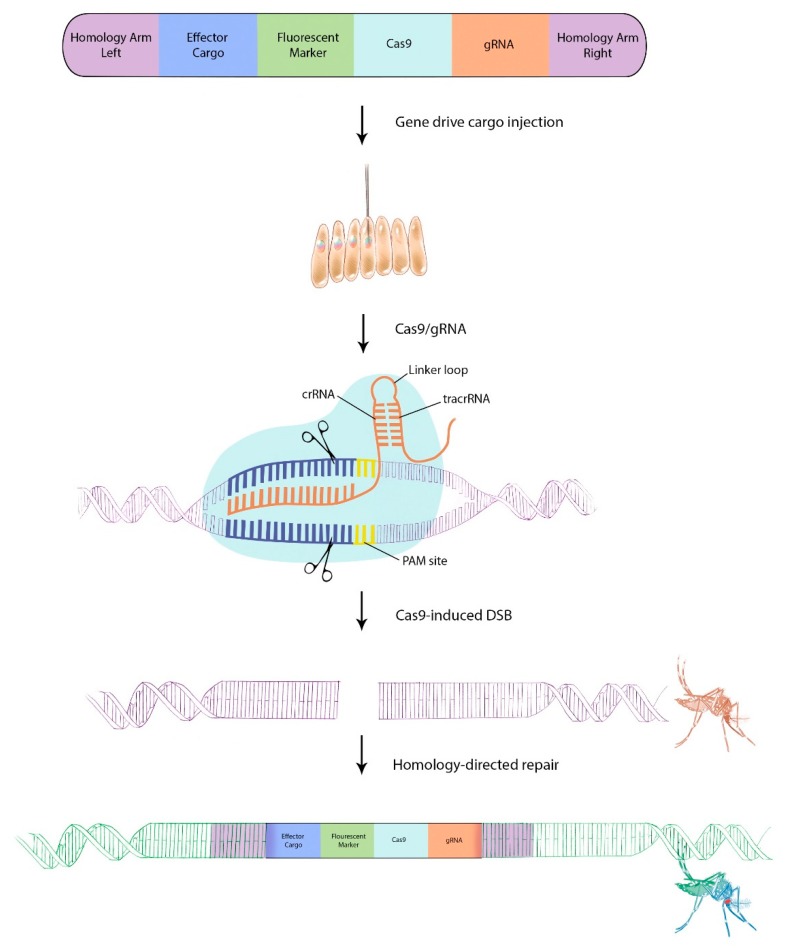
Generalized flow chart for the establishment of a one-component CRISPR/Cas9-based gene drive system in *Ae. aegypti*. Antiviral effector cargo, when expressed alongside the necessary components of the CRISPR/Cas9 system, can be driven into mosquito populations. Such a gene drive construct would contain (1) flanking homology arms that are complementary to the CRISPR/Cas9 target site in the mosquito genome, (2) the antiviral effector under control of a tissue-specific promoter, (3) a discernable marker (such as a fluorescent protein under control of a photoreceptor-specific promoter), (4) the Cas9 enzyme under control of a germline-specific promoter, and (5) a gRNA under control of an RNA polymerase III (U6) promoter. The construct is injected into pre-blastoderm embryos for site-specific germline integration. Once expressed, the gRNA forms a complex with the Cas9 enzyme and guides it to complementary sequences upstream of a PAM site. The Cas9 enzyme then induces a DSB 3–5 bp upstream of the PAM site. If the cell uses HDR to repair the DSB, the homologous sequence in the donor plasmid will be used as template to repair the DSB. The G1 offspring then represents transgenic mosquitoes, which are capable of gene driving by targeting the wild-type allele with CRISPR/Cas9 and repairing the cleavage though HDR, thereby inserting the entire gene drive system including the antiviral effector cargo.

The first proof-of-principle study that CRISPR/Cas9 can be used as a synthetic gene drive system in mosquitoes was shown for *Anopheles stephensi* in 2015 [77]. Similar to HEG, the CRISPR/Cas9 machinery along with the appropriate gRNA can be designed as a gene drive system to be allele-specific and inherited by subsequent generations (Figure 3). A CRISPR/Cas9-based gene drive construct must generally encode, at a minimum: (1) Flanking homology arms serving as a DNA template complementary to the mosquito genome to facilitate HDR-mediated knock-in of the transgenic cargo, (2) the Cas9 enzyme, under control of a germline-specific promoter, and (3) an endogenously expressed gRNA [78]. Recently, a study examined the efficiency of 12 *Ae. aegypti* RNA polymerase III U6 promoters, four of which, including the U6 promoter initially described by Konet and colleagues (2007), were found to facilitate efficient CRISPR/Cas9 mediated genome editing [79,80].

There are several challenges in regard to the design of such a gene drive construct. An optimal genome locus must be identified to allow stable, site-specific insertion of the gene drive system. This locus should be highly conserved among the diverse populations of *Ae. aegypti* mosquitoes to facilitate robust spread of an antiviral effector gene. Once such a stable genome locus has been identified, the efficiency of various gRNAs to target this locus needs to be evaluated. Because Cas9 efficiency has been reported to vary significantly between different target sites, the optimal gRNA sequence should be identified in a comparative assay. If the targeted locus is not conserved among individuals of the target population or is prone to mutations or indel formation, the selected gRNA will no longer be complementary to the target sequence. Consequently, the CRISPR drive (along with the antiviral effector) can no longer be inserted into the homologous allele; the gene drive system stalls, meaning that the antiviral effector will no longer be passed on to subsequent generations. In addition to naturally occurring sequence variation that could prevent a sequence homology dependent gene drive from spreading, resistance to the gene drive system could also develop during the repair process of the genomic DNA following its cleavage by Cas9. If the DNA repair undergoes homologous recombination with the gene drive containing DNA template, the drive system will continue spreading through the population. If, however, the DNA repair undergoes NHEJ, the CRISPR/Cas9 target site becomes modified and is no longer recognized by the Cas9/gRNA complex. In addition, maternal effect, when the phenotype of the offspring is influenced by the mother’s genotype or phenotype, can result in drive-resistant allele formation [69]. Generally, sequence homology dependent gene drive systems are sensitive to the development of drive-resistant alleles [81].

Several strategies have been developed to address these problems. For example, multiplexing the CRISPR/Cas9 construct by using more than one gRNA has been shown to efficiently result in multiple simultaneous gene disruptions [82], and experiments with *D. melanogaster* have shown that gRNA multiplexing significantly reduces resistance allele formation rates [83]. Since the efficiency of the CRISPR/Cas9-mediated gene drive heavily relies on the timing and the level of expression of its components in specific tissues of the insect, the choice of optimal promoters has to be regarded as one of the most critical aspects of the overall gene drive design. Restricting Cas9 expression to the germline has been shown to improve HDR rates [69,84]. Recently, the efficiency of several germline promoters have been thoroughly tested in *Ae. aegypti* [82]. This then led to the generation of several *Ae. aegypti* lines, which stably express Cas9 in the germline. Furthermore, using an optimal polymerase III promoter for gRNA expression, and the *exuperentia* or *ubiquitin L40* promoter for Cas9 expression, Li and colleagues developed the first two-component CRISPR/Cas9-based gene drive (split drive) system for *Ae. aegypti*, based on the “Copy Cat” approach. This split drive system was inherited with a rate exceeding 90% over several generations [80,82].

## 6. *Aedes aegypti* Population Replacement Models

As more and more laboratory and semi-field cage studies simulate mosquito population modification strategies, computer models will always be necessary to predict the feasibility of these novel approaches and to help determine the number of insects required to be released to drive a desired transgene to fixation in a target population. Various gene drive model platforms exist, including SkeeterBuster, SLiM 2/3, and MGDrivE, although they vary in flexibility [85,86,87,88].

SkeeterBuster which has been validated in Iquitos, Peru, takes into account how field conditions, including adult dispersal distances and larval habitat availability, may affect the *Ae. aegypti* population dynamics after mosquito releases [83]. The model can estimate F_st_ values, or the level of genetic differentiation between subpopulations, as well as the time period required for allele fixation or extinction and the number of transgenic mosquitoes necessary for desired population replacement outcomes. However, the model is limited by the number of genetic control strategies that can be simulated. SLiM provides increased user flexibility and can be scripted by the user to simulate many evolutionary scenarios, including CRISPR/Cas9-mediated gene drive [85]. SLiM can estimate the time period to fixation while accounting for fitness costs associated with the gene drive system, homing efficiency, and mating preferences [86,87]. Because this model is designed for a more general audience, however, it does not account for complex ecological parameters that could impact the success of a release strategy for mosquito populations. A third model is the versatile MGDrivE (Mosquito Gene Drive Explorer) model [88]. It can be used to estimate the efficacy of a variety of gene drive systems given a wide variety of parameters, which could be expanded to both population replacement and population suppression studies. The key components of the model include genetic inheritance (sex-specific homing efficiency and homing resistance rates), mosquito life history (fitness cost parameters measured at each life stage, including egg production, mortality risk, and longevity), and landscape (target population size and size of geographic target region). From these user defined variables, the model can estimate required release numbers with respect to sex ratios and time periods required for g.o.i. fixation.

To date, no cage studies have been implemented to test gene drives linked to anti-pathogen effector genes in *Ae. aegypti*. These studies will be a crucial next step to validate the method under more realistic field conditions. Such experiments will be especially useful when assessing the likelihood of resistance that may evolve against the gene drive as well as against the anti-pathogen effector because of the repeated selection pressure. In *An. gambiae* population suppression studies, for example, mutations that resisted the CRISPR/Cas9-based gene drive emerged after 25 generations [89]. The SLiM model has also been used to estimate the rate of g.o.i. fixation in regard to high and low rates of drive-resistance allele formation [83]. These simulations estimate that when using a multiplexed Cas9 gene drive approach with rates of low resistance, the drive can reach fixation already within 20 subsequent generations; however, the authors argue that such an approach may not be sufficient to robustly convert wild populations [83]. Indoor cage trials will be necessary to simulate the rates of gene-drive resistance, which can then be used in the outlined models to estimate the required release numbers and time period to achieve g.o.i. fixation.

Regardless, before transgenic *Ae. aegypti* transition to field conditions outside the laboratory, there is already growing concern that such a gene drive could invade non-target insect populations or self-propagate out of control. Therefore, “confinable” gene drives have been investigated and modeled using the MGDrivE platform [80]. These systems include split drives, whereby a two-component system is established with the trans-acting Cas9 and the gRNA segregating independently from one generation to the next. Mosquitoes expressing either (but not both) of these constructs do not exhibit gene drive; however, crossing mosquitoes that harbor the gRNA component with those that harbor the Cas9 expression cassette would result in progeny that exhibit gene drive activity with varying inheritance patterns (estimated to range from 71 to 100%), depending on the efficiencies of the gRNA and Cas9 promoters [80]. MGDrivE modeling estimates that 10 weekly releases of 10,000 homozygous males expressing the split gene drive into a population of 10,000 mosquitoes could drive the transgene to fixation within ~8 months. This could last >4 years, given an estimated 10% fitness cost associated with the transgene [80]. The split drive is expected to be confinable: the gRNA/anti-pathogen allele is estimated to reach 15% allele frequency in nearby populations (compared to 50% in the absence of a split gene drive) after 10 releases, before being gradually eliminated because of fitness costs. Importantly, the release of wild-type males can reverse the action of the split gene drive system if necessary [80].

## 7. Conclusions

Genetic control strategies targeting *Ae. aegypti* represent a novel set of tools for vector control programs. Population modification techniques in *Ae. aegypti* are becoming especially attractive now that a range of tissue-specific promoters have been identified, antiviral effector genes have been optimized and tested under laboratory conditions, and efficient gene drive systems are being developed. However, there is still much progress to be made. No antiviral effector gene has been tested so far in the context of gene drive, and to date, there have been no field studies conducted to validate the population replacement strategies. Laboratory and computer simulations that calculate fitness costs associated with the transgene and release rates necessary for transgene fixation in a given population will need to be assessed before genetic control strategies can be robustly implemented under field conditions. These models can also estimate the long-term effects of gene drive systems, including the potential for mutations and resistance to emerge in the mosquito vector as well as the probability of transgene “spill-over” into neighboring populations. Before gene drive studies can transition to real-world applications, transparency, safety, and standardized quality control methods need to be implemented. Several publications outline standard operating procedures and containment guidelines for genetically modified organisms in the context of gene drive [90,91,92]. Community engagement and safety regulations will be as crucial as scientific rigor before gene drive technologies can be applied to mitigate the prevalence of arboviruses in the field.

## Figures and Tables

**Table 1:** Transgenic *Ae. aegypti* engineered (to date) to be resistant to arbovirus infections.

Study	Transgenic Strategy	Virus Targeted	Method	Promoter	Prevalence of Disseminated Infections in Transgenics
Franz et al. 2006 [25]	^†^ IR triggering siRNA antiviral pathway	DENV2	*mariner Mos1*	^††^ *AeCPA*	0% (14 dpi ^§^)
Franz et al. 2014 [27]					
Mathur et al. 2010 [26]	IR triggering siRNA antiviral pathway	DENV2	*mariner Mos1*	^‡^ *Ae30K b*	0% (saliva, 14 dpi)
Mishra et al. 2016 [32]	Antiviral hammerhead ribozymes	CHIKV 181/25	*piggyBac*	^‡‡^*AetRNA^val^* Pol III	0% (7 dpi)
Jupatanakul et al. 2017 [33]	Overexpression of components in the JAK/STAT pathway	DENV2/4	*piggyBac*	* *AeVg1*	≥43% (14 dpi)
Yen et al. 2018 [35]	Synthetic RNAs targeting virus genome, triggering miRNA antiviral immunity	DENV3/CHIKV	*mariner Mos1*	** *AePUb/AeCPA*	~10% (DENV3, 21 dpi)
					~10–50% (CHIKV, 6 dpi)
Buchman et al. 2019 [36]	Synthetic RNAs targeting virus genome, triggering miRNA antiviral immunity	ZIKV	*piggyBac*	*AeCPA*	0% (14 dpi)
Buchman et al. 2019 [37]	Broadly neutralizing single chain antibody	DENV1-4	*piggyBac*	*AeCPA*	0% (14 dpi)

^†^ IR = inverted-repeat construct; ^††^ *AeCPA* = *Ae. aegypti carboxypeptidase A* promoter; ^‡^ *Ae30K b* = *Ae. aegypti Aegyptin* promoter; ^‡‡^ *AetRNA^val^* Pol III = *Ae. aegypti* RNA polymerase III valine promoter; * *AeVg1* = *Ae. aegypti vitellogenin 1* promoter; ** *AePUb* = *Ae. aegypti Polyubiquitin* promoter; ^§^ dpi = days post-infection.
